# The complete chloroplast genome of *Toxicodendron griffithii*

**DOI:** 10.1080/23802359.2020.1768931

**Published:** 2020-05-28

**Authors:** Yongjie Li, Yiyun Tang, Tao Wu

**Affiliations:** a Institute of Economic Forest, Yunnan Academy of Forestry and Grassland, Kunming, Yunnan, China; b Woody Oil Industry Research Institute of Nujiang Prefectrue, Lushui, Yunnan, China; c Yunnan Laboratory for Conservation of Rare, Endangered and Endemic Forest Plants, Public Key Laboratory of the State Forestry Administration, Kunming, Yunnan, China; d Yunnan Provincial Key Laboratory of Cultivation and Exploitation of Forest Plants, Kunming, Yunnan, China

**Keywords:** *Toxicodendron griffithii*, chloroplast genome

## Abstract

The whole chloroplast (cp) genome sequence of *Toxicodendron griffithii* has been characterized from Illumina pair-end sequencing. The complete cp genome was 159,613 bp in length, containing a large single-copy region (LSC) of 87,722 bp and a small single-copy region (SSC) of 18,911 bp, which were separated by a pair of 26,490 bp inverted repeat regions (IRs). The genome contained 132 genes (113 unique), including 87 protein-coding genes (80 unique), 37 tRNA genes (29 unique), and 8 rRNA genes (4 unique). The overall GC content of *T. griffithii* cp genome is 37.94%. Phylogenetic analysis of 14 chloroplast genomes within the family Anacardiaceae suggests that *T. griffithii* is closely related to genus *Rhus* and genus *Pistacia.*

*Toxicodendron griffithii* (J. D. Hooker) Kuntze belongs to the genus *Toxicodendron* in Anacardiaceae is a deciduous tree species with globose drupe, thick, and waxy mesocarp, narrowly scattered in SW China (Guizhou and Yunnan) and NE India (Darjeeling) (Min and Anders 2009). Urushiol is a unique biochemical component in Anacardiaceae, mainly from *Toxicodendron*, which has excellent biological activities in anti-oxidation, anti-tumor, and anti-virus and then has great values in the development of pharmaceutical and industrial production (Kim et al. 2014; Lee et al. 2018). The native populations of the species have dramatically declined in the past decades. Hence, it is important to set up appropriate conservation strategies to conserve the genetic diversity of the species.

We sampled a natural population with fresh, healthy leaves of *T. griffithii* from Chenggan county of Yunnan, China (N 26°11′57″, E 98°55′34″). A single individual was selected for total genomic DNA isolation using modified CTAB protocol (Yang et al. 2014). Voucher specimen (Wu180801) was deposited in the Herbarium of Yunnan Academy of Forestry and Grassland, Kunming, China.

A shotgun library was prepared and sequenced using the Illumina Hiseq 2500 (Illumina, CA, USA). Approximately, 3 Gb raw data of 150 bp paired-end reads were obtained. The raw reads were filtered to obtain high-quality clean reads by using NGS QC Toolkit_v2.3.3 with default parameters (Patel and Jain 2012). Plastomes were assembled as in Yang et al. (2014). The genome was automatically annotated using the Dual Organellar GenoMe Annotator (DOGMA) tool (Wyman et al. 2004), then adjusted, and confirmed with Geneious 9.1 (Kearse et al. 2012). The annotated genomic sequence was then deposited into GenBank with the Accession Number MT269874.

The complete chloroplast genome of *T. griffithii* is 159,613 bp in length, containing a pair of inverted repeats (IRs) of 26,490 bp, a small single-copy (SSC) of 18,911 bp and a large single-copy (LSC) of 87,722 bp. The overall GC content of the chloroplast genome is 37.94%. A total of 113 genes in the chloroplast genome were annotated, including 80 protein-coding genes, 29 tRNA genes, and 4 rRNA genes. To ascertain the phylogenetic status of *T. griffithii*, the complete chloroplast genome sequences of 14 species from the family Asparagaceae were aligned using MAFFT (Katoh and Standley 2013) and a maximum likelihood tree was reconstructed with RAxML8 (Stamatakis 2014), which revealed that genus *Toxicodendron* were closely related to genus *Rhus* and genus *Pistacia* than other genera (Figure 1).

**Figure 1.**
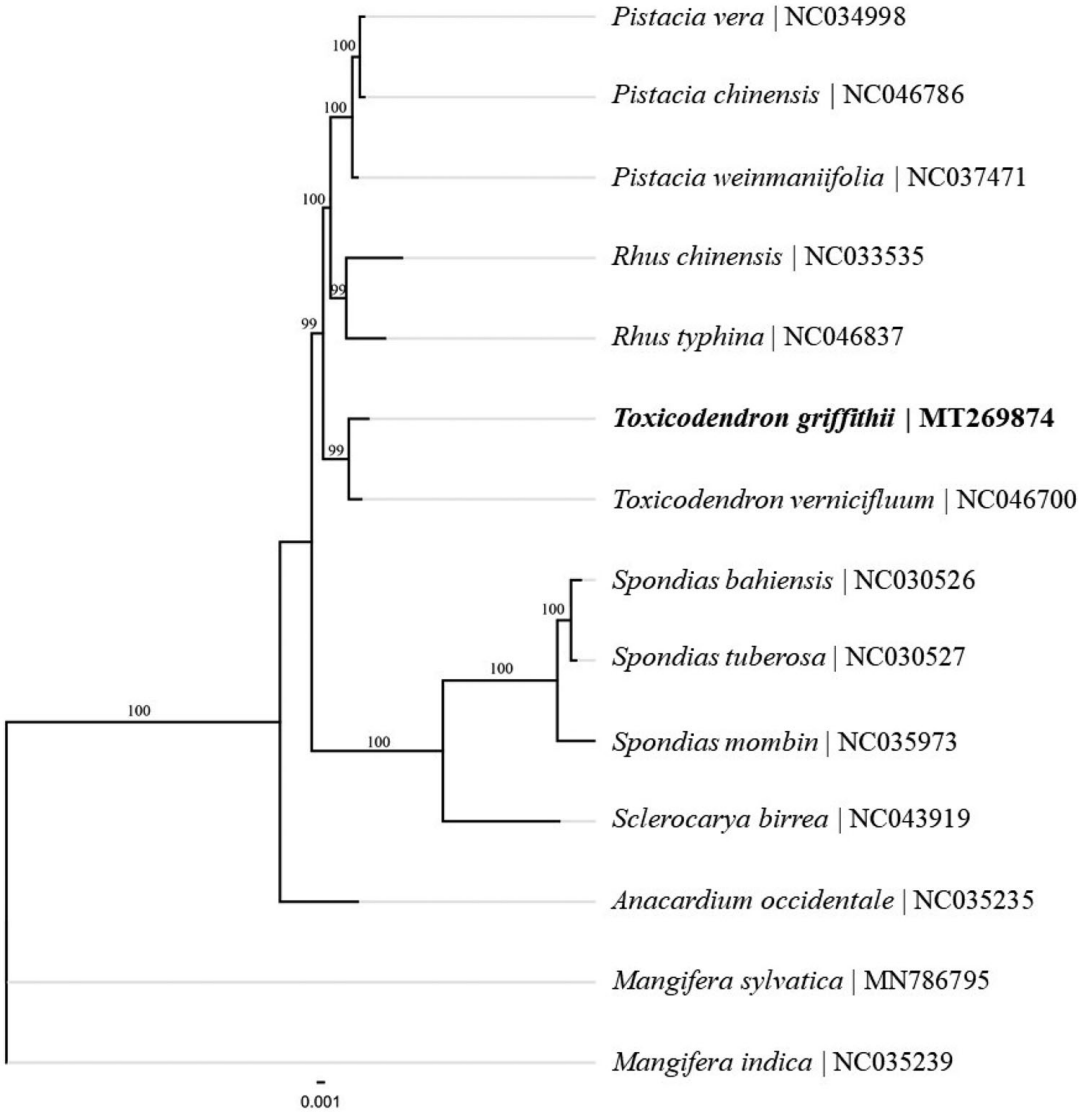
Phylogenetic relationships of 14 species from the family Asparagaceae based on the complete chloroplast genome sequences. Bootstrap percentages are indicated for each branch.

The newly characterized complete chloroplast genome of *T. griffithii* will provide essential resources for further study on the evolution and genetic diversity.

## Data Availability

The data that support the findings of this study are openly available in GenBank at https://www.ncbi.nlm.nih.gov/nuccore/?term=MT269874, the accession number is MT269874.
